# Changes in Elastic Moduli of Fibrin Hydrogels Within the Myogenic Range Alter Behavior of Murine C2C12 and Human C25 Myoblasts Differently

**DOI:** 10.3389/fbioe.2022.836520

**Published:** 2022-05-20

**Authors:** Janine Tomasch, Babette Maleiner, Philipp Heher, Manuel Rufin, Orestis G. Andriotis, Philipp J. Thurner, Heinz Redl, Christiane Fuchs, Andreas H. Teuschl-Woller

**Affiliations:** ^1^ Department Life Science Engineering, University of Applied Sciences Technikum Wien, Vienna, Austria; ^2^ The Austrian Cluster for Tissue Regeneration, Vienna, Austria; ^3^ Ludwig Randall Centre for Cell and Molecular Biophysics, King’s College London, Guy’s Campus, London, United Kingdom; ^4^ Institute of Lightweight Design and Structural Biomechanics, TU Wien, Vienna, Austria; ^5^ Ludwig Boltzmann Institute for Traumatology, The Research Center in Cooperation with AUVA, Vienna, Austria; ^6^ Wellman Center for Photomedicine, MGH, Boston, MA, United States; ^7^ Harvard Medical School, Boston, MA, United States

**Keywords:** skeletal muscle tissue engineering, fibrin, biomaterials, mechanobiology, myogenesis

## Abstract

Fibrin hydrogels have proven highly suitable scaffold materials for skeletal muscle tissue engineering in the past. Certain parameters of those types of scaffolds, however, greatly affect cellular mechanobiology and therefore the myogenic outcome. The aim of this study was to identify the influence of apparent elastic properties of fibrin scaffolds in 2D and 3D on myoblasts and evaluate if those effects differ between murine and human cells. Therefore, myoblasts were cultured on fibrin-coated multiwell plates (“2D”) or embedded in fibrin hydrogels (“3D”) with different elastic moduli. Firstly, we established an almost linear correlation between hydrogels’ fibrinogen concentrations and apparent elastic moduli in the range of 7.5 mg/ml to 30 mg/ml fibrinogen (corresponds to a range of 7.7–30.9 kPa). The effects of fibrin hydrogel elastic modulus on myoblast proliferation changed depending on culture type (2D vs 3D) with an inhibitory effect at higher fibrinogen concentrations in 3D gels and *vice versa* in 2D. The opposite effect was evident in differentiating myoblasts as shown by gene expression analysis of myogenesis marker genes and altered myotube morphology. Furthermore, culture in a 3D environment slowed down proliferation compared to 2D, with a significantly more pronounced effect on human myoblasts. Differentiation potential was also substantially impaired upon incorporation into 3D gels in human, but not in murine, myoblasts. With this study, we gained further insight in the influence of apparent elastic modulus and culture type on cellular behavior and myogenic outcome of skeletal muscle tissue engineering approaches. Furthermore, the results highlight the need to adapt parameters of 3D culture setups established for murine cells when applied to human cells.

## Introduction

Skeletal muscle not only gives us the ability to move but also has numerous other essential functions in the human body. Although muscle tissue has a high regenerative capacity, when more than 20% of the tissue of a particular muscle is lost, healing of the tissue is substantially hampered (Jana, Levengood, and Zhang 2016). Besides volumetric muscle loss due to trauma or muscular diseases, muscle wasting is a considerable socioeconomical burden affecting 561 million people worldwide. Therefore and since autologous muscle grafts harbor the risk of donor-site morbidity and additional impairment of quality of life, the need for strategies to generate *ex vivo* functional muscle tissue is evident (Manring et al., 2014; Grasman et al., 2015).

Shortly after the boom in tissue engineering (TE), a new wave emerged within this field: it became apparent that the mechanical properties of scaffold materials or the cell microenvironment and related mechanotransduction are of utmost importance when trying to engineer tissue. Although the science of mechanotransduction and biomechanics is not new, it gained substantial attention through the TE field. Numerous reviews and publications in top-tier peer-reviewed journals are proof (Martino et al., 2018; Chaudhuri et al., 2020). Biomaterial and TE research not only focuses on the identification of novel biomaterials for TE purposes or their tuneability, but also on how the material properties can influence cellular behavior or tissue formation. In general, most cell types are capable of self-organization into tissues and synthesizing as well as modifying their extracellular environment. This can be achieved by different processes, as reviewed by Sthijns *et al*., who highlighted the importance of organization in a three dimensional environment for cell fate decisions and maturation (Werner, Vu, and Rink 2017). It is well accepted that providing the right microenvironment of biophysical and biochemical cues highly influences the outcome in TE constructs (Sthijns, Lapointe, and Van Blitterswijk 2019).

It has long been known that in skeletal muscle tissue engineering (SMTE) structural and mechanical cues in the respective scaffold biomaterial, such as micro-architecture and stiffness, guide and induce muscle differentiation and are pivotal for the myogenic outcome (Engler et al., 2004; Gilbert et al., 2010; Hinds et al., 2011; Romanazzo et al., 2012). To name just one example, culture on micropatterned soft gels with substrate stiffness in the range of 8–11 kPa generated striated myotubes in contrast to stiffer substrates (Engler et al., 2004). These findings are in accordance with several other studies that found that culture on softer matrices is more beneficial for myogenesis compared to stiffer ones (Engler et al., 2004; Hu et al., 2011; Romanazzo et al., 2012; Gribova et al., 2013). The range of substrate stiffness analyzed in most studies, however, varies over several orders of magnitude (from 1 kPa to several MPa), reducing their biological relevance, as minor changes in biomaterial modulus can already elicit substantial changes in the embedded biological system. The premise of most studies analyzing the effect of substrate stiffness, and SMTE approaches in general, is the use of scaffolds with mechanical properties close to the native tissue. In this regard, however, the stiffness of skeletal muscle depends on age and health status and also species (Table 1), and this has to be taken into account for designing patient-specific regenerative approaches. Thus, when translating findings from different studies using murine cells to SMTE approaches with human cells, the same outcomes cannot be expected. Furthermore, the choice of method for mechanical characterization [e.g. tensile testing or atomic force microscopy (AFM)] may influence the results due to the load case applied and the length-scale at which the method is applied.

**TABLE 1:** Overview of mechanical properties of skeletal muscle in mice and humans.

Mouse							Human				
**Source**			**Modulus/Stiffness**		**Method**		**Source**		**Modulus/Stiffness**	**Method**	
***Single fibers***											
	Healthy	12 ± 4 kPa			AFM (indentation)	Engler et al. (2004)					
	dystrophic	18 ± 6 kPa			AFM (indentation)	Engler et al. (2004)	** **				
adult	Intact	0.4 ± 0.1 kPa			AFM (indentation)	Lacraz et al. (2015)	** **				
	damaged	2.3 ± 0.4 kPa			AFM (indentation)	Lacraz et al. (2015)	young		∼8 kPa/μm^a^	force transducer (stretch-relax)	Noonan et al. (2020)
old	Intact	1.9 ± 0.3 kPa			AFM (indentation)	Lacraz et al. (2015)		old	∼12 kPa/μm^a^	force transducer (stretch-relax)	Noonan et al. (2020)
	damaged	10.4 ± 1.6 kPa			AFM (indentation)	Lacraz et al. (2015)		healthy	28.2 ± 3.3 MPa	force transducer (stretch-relax)	Lieber et al. (2003)
										
	Healthy	∼7 kPa			force transducer (stretch-relax)	Meyer and Lieber (2011)		spastic	55 ± 6.6 MPa	force transducer (stretch-relax)	Lieber et al. (2003)
	Healthy	∼40 kPa			force transducer (stretch-relax)	Meyer and Lieber (2011)		healthy	462.5 ± 99.6 MPa	force transducer (stretch-relax)	Lieber et al. (2003)
** **								spastic	111.2 ± 35.5 MPa	force transducer (stretch-relax)	Lieber et al. (2003)
***Whole muscle***											
** **							young	male	292 ± 36 N/m^b^	MyotonPRO (damped natural oscillation)	Agyapong-Badu et al. (2016)
** **								female	233 ± 35 N/m^b^	MyotonPRO (damped natural oscillation)	Agyapong-Badu et al. (2016)
** **							old	male	328 ± 29 N/m^b^	MyotonPRO (damped natural oscillation)	Agyapong-Badu et al. (2016)
** **								female	311 ± 42 N/m^b^	MyotonPRO (damped natural oscillation)	Agyapong-Badu et al. (2016)

AFM, atomic force microscopy.

a Passive elastic modulus at a sarcomere length of 2.5 µM.

b Stiffness.

Although researchers have looked at different geometries, (bio)-materials and stiffness (Lam et al., 2009; Bajaj et al., 2011; Rizzi et al., 2012; Ostrovidov et al., 2014; Heher et al., 2015), there is still no consensus about optimal material properties to foster myogenesis in order to engineer muscle-like tissue in the SMTE field. This stems from the fact that many studies either solely look at 2D differentiation of TE muscle or 3D setups. Few are comparing both and results on the effects on myogenesis from such comparisons are often different. One example is that in 2D, murine myoblasts only aligned in grooves less than 100 µm wide, whereas in 3D constructs, these cells only aligned in larger grooves of 200 µm (Hume et al., 2012), highlighting the differences of cell responses depending on 2D *vs* 3D culture.

In a previous study, our group used 3D fibrin scaffolds for the generation of muscle-like structures through application of tensile stress onto myoblasts (Heher et al., 2015). We chose fibrin as a cell carrier, since it has proven highly suitable for SMTE approaches in the past (Huang et al., 2005; Beier et al., 2006; Matsumoto et al., 2007; Bian and Bursac 2009; Lam et al., 2009; Wang, Shansky, and Vandenburgh 2013; Heher et al., 2015) as we further elaborated on in a follow-up review (Maleiner et al., 2018). To the best of our knowledge, despite its popularity, there are no studies directly comparing cellular behavior in 2D *vs* 3D in fibrin in SMTE. Therefore, this aspect of 2D *vs* 3D cultures of myoblasts on or within fibrin with varying stiffness will be analyzed within this study.

A further issue tackled in this study is the transferability of the material’s influence on myogenesis in murine to human models. The most frequently used myoblast cell line used in SMTE is the murine myoblast cell line C2C12, as it constitutes a fast, reliable and low-cost approach. Without question, the data generated and contributions to the field are essential but, ultimately, the goal is to engineer tissue that is transplantable into humans or serves as a human model of muscle tissue for drug screens, disease modelling or simulating acute pathologies such as trauma. Thus, efforts have been made to engineer skeletal muscle-like tissue using various human cell types, ranging from satellite cells to induced pluripotent stem cells and myoblasts (Tonlorenzi et al., 2007; Ostrovidov et al., 2015; Maffioletti et al., 2018; Maleiner et al., 2018; Rao et al., 2018). Successful translation of concepts developed in murine SMTE approaches to the use of human cells requires similar responses of human cells to these specific environments. In order to obtain this information, studies applying cell types with similar myogenic potential derived from different species in the same culture setups are required. We want to achieve this through comparison of the widely used murine myoblast cell line C2C12 to the human myoblast cell line C25 (Thorley et al., 2016), which has gained increasing importance in human skeletal muscle models. Noteworthy, transcriptomic analysis showed that immortalization of C25 myoblasts did not overtly affect their behavior throughout myogenesis (Thorley et al., 2016), which ensures comparability of the line with the murine C2C12 myoblasts.

With this study, we want to shed light on the impact of culture conditions in fibrin hydrogels on a well-established murine myoblast cell line, C2C12, and the human C25 myoblast cell line, by answering the following questions: *1*) How do subtle variations in fibrin hydrogel stiffness within a defined range, proposed to be most compatible with myogenesis, affect murine and human myoblasts?; *2*) Does the culture type, namely 2D vs 3D, affect myogenesis differentially in myoblasts from these two species?; and *3*) How does the combination of stiffness and culture type influence myogenic differentiation? These are key questions in the SMTE field, and therefore this comparison of species and other essential differentiation cues has potential to contribute to unresolved issues in the SMTE field advancing it a step further.

## Materials and Methods

If not indicated otherwise, all chemicals and reagents were purchased from Sigma Aldrich (Vienna, Austria) and were of analytical grade.

### Cell Culture

Two different myoblast cell lines were used for this study. The murine line C2C12 (American Type Culture Collection, Manassas, United States) was cultured in Dulbecco’s modified Eagle’s medium high glucose (DMEM-HG; Life Technologies, Carlsbad, CA, United States), supplemented with 10% fetal calf serum (v/v) (GE Healthcare, Buckinghamshire, United Kingdom), 1% penicillin/streptomycin (v/v) (Lonza, Basel, Switzerland) and 1% L-glutamine (v/v) (Lonza, Basel, Switzerland). The human line C25 (kind gift from Peter Zammit, King’s College, London, United Kingdom) was cultured in skeletal muscle cell growth medium (PromoCell, Heidelberg, Germany), supplemented with the skeletal muscle cell growth medium supplement kit (PromoCell, Heidelberg, Germany) with final concentrations of 50 μg/ml bovine fetuin, 10 ng/ml recombinant human epidermal growth factor, 1 ng/ml recombinant human basic fibroblast growth factor, 10 μg/ml recombinant human insulin, 0.4 μg/ml dexamethasone, and 20% fetal calf serum (v/v), 1% penicillin/streptomycin (v/v) and 1% L-glutamine (v/v). Those media will be referred to as growth medium (GM). For expansion, cells were cultured in standard cell culture dishes (37°C, 5% CO_2_) and sub-cultured at 70% confluence to avoid induction of differentiation. Differentiation media (DM) consisted of DMEM-HG, supplemented with 3% horse serum (v/v), 1% penicillin/streptomycin (v/v) and 1% L-glutamine (v/v) for C2C12 cells and skeletal muscle cell differentiation medium (PromoCell, Heidelberg, Germany), supplemented with recombinant human insulin (10 μg/ml), 1% penicillin/streptomycin (v/v) and 1% L-glutamine(v/v) for C25 cells.

### Preparation of Fibrin Hydrogels for Cell-Based Experiments

**FIGURE 1:**
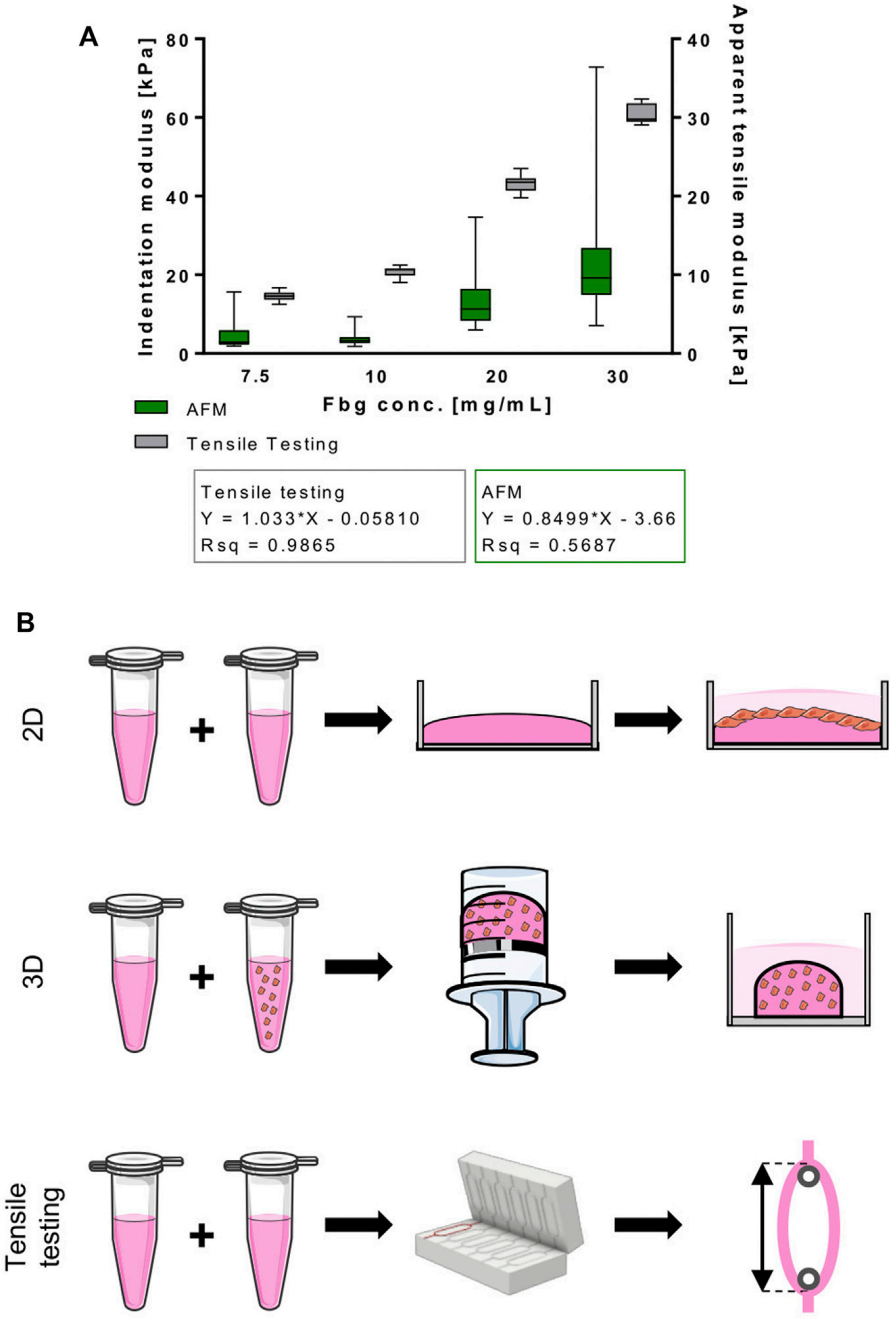
Normalized stiffness of fibrin hydrogels used in this study correlates directly with fibrinogen content. **(A)** Indentation and apparent tensile modulus [kPa] of fibrin hydrogels with different fibrinogen concentrations [mg/ml] acquired through AFM indentation and tensile testing, respectively. Data is shown as box and whiskers (min to max); *N* ≥ 2, *n* ≥ 6 for tensile testing and *N* = 2, *n* = 2 AFM. **(B)** Hydrogel types used in this study. 2D: cells were seeded on top of fibrin-coated wells of cell culture well-plates; 3D: cells were incorporated into fibrin hydrogels that were polymerized in 2 ml syringes and transferred to cell culture well-plates afterwards; tensile testing: fibrin hydrogels were cast in a ring shape and mounted onto spools for tensile testing.

To investigate the effect of substrate stiffness and architecture on myoblasts, cells were either cultured on fibrin-coated well-plates (referred to as 2D) or encapsulated in fibrin hydrogels (referred to as 3D) using the clinically approved Tissucol Duo 500 5.0 ml Fibrin Sealant (Baxter Healthcare Corp., Deerfield, United States) (Figure 1B).

For the 2D culture setups, fibrin coating was generated by mixing fibrinogen and thrombin in a 1:1 ratio. Different fibrinogen concentrations were used to create hydrogels with different mechanical properties, while the final thrombin concentration was 1 U/mL for all hydrogels. Fibrinogen was diluted from a 78.5 mg/ml stock in the respective GM. A thrombin stock of 500 U/ml was diluted in 40 mM CaCl_2_ to a working solution of 4 U/ml and further diluted in GM. Polymerization of fibrin coating was achieved after 30 min at 37°C prior to cell seeding.

For the 3D culture setups, different fibrinogen concentrations were used with a constant final concentration of 1 U/ml thrombin. To encapsulate cells in the hydrogels, the thrombin working solution was diluted with a cell suspension (in GM). Thrombin+cells and fibrinogen were mixed in a 1:1 ratio and polymerized in 2 ml syringes, of which the cap had been cut off, for 30 min at 37°C.

### Cell Preparation for Proliferation Assay

For analysis of proliferative behavior, hydrogels were generated as described above. For the 2D setup, fibrin coatings with a final volume of 300 µl in 24-well plates with the different fibrinogen concentrations (5 mg/ml fibrinogen, 10 mg/ml fibrinogen and 20 mg/ml fibrinogen) were created. The hydrogels were seeded with 1 × 10^5^ cells in their respective GM. Proliferative behavior in a 3D environment was analysed by encapsulating 1 × 10^5^ cells in fibrin hydrogels. 3D fibrin hydrogels had a final volume of 300 μL at different fibrinogen concentrations (5 mg/ml fibrinogen, 10 mg/ml fibrinogen and 20 mg/ml fibrinogen).

Cells were cultured in their respective GM, supplemented with the fibrinolysis inhibitor aprotinin at 100 KIU/ml (Baxter Healthcare Corp., Deerfield, United States) for 4 days. A medium exchange with GM was performed on day 2 and DNA quantification on day 0 (D0), day 2 (D2) and day 4 (D4).

### Cell Preparation for Differentiation Assay

For analysis of differentiation, hydrogels were generated as described above. For the 2D setup, fibrin coatings with a final volume of 400 µl in six-well plates with the different fibrinogen concentrations of 7.8 mg/ml fibrinogen, 11.67 mg/ml fibrinogen and 19.42 mg/ml fibrinogen corresponding to apparent tensile moduli of 8, 12 and 20 kPa, respectively, as assessed by tensile mechanical testing, were created. The hydrogels were seeded with 5 × 10^5^ cells in their respective GM on D0 of the experiment. Differentiation in a 3D environment was analyzed by encapsulating 2.4 × 10^6^ cells in fibrin hydrogels on D0. Fibrin hydrogels had a final volume of 300 µl and different fibrinogen concentrations (7.8 mg/ml fibrinogen, 11.67 mg/ml fibrinogen and 19.42 mg/ml fibrinogen (corresponding to tensile moduli of 8, 12 and 20 kPa, respectively as assessed by tensile mechanical testing). Cells were cultured in media supplemented with 100 KIU/ml aprotinin for a total of 5 days. GM was replaced by DM on day 1 (D1) to induce differentiation and partially (1:1) exchanged with GM on day 3 (D3).

### Preparation of Cell-Free Fibrin Hydrogels for Mechanical Characterization

To assess the effect of fibrinogen content on fibrin hydrogels’ apparent tensile moduli, hydrogels were cast in ring-shaped scaffolds, as previously described (Heher et al., 2015) (Figure 1B). Briefly, scaffolds were cast by mixing 250 µl fibrinogen and 250 µl thrombin to achieve final concentrations of 7.5 mg/ml fibrinogen, 10 mg/ml fibrinogen, 20 mg/ml fibrinogen and 30 mg/ml fibrinogen with a constant concentration of 1 U/ml thrombin. Hydrogels were polymerized in custom-made POM molds for 30 min at 37°C and then equilibrated in phosphate-buffered saline (PBS) overnight. Fibrin hydrogels for AFM microindentation experiments were cast according to the procedure for 2D fibrin coatings (Figure 1B) and stored in PBS overnight.

### Mechanical Characterization of Fibrin Scaffolds

Tensile mechanical testing: cell-free scaffolds were mounted on two spools for tensile testing with a Zwick BZ2.5/TN1S uniaxial material testing machine (Zwick GmbH & Co., KG, Ulm, Germany) (as described in (Heher et al., 2015)) 1 day after casting. Briefly, samples were strained to failure at a rate of 20 mm/min at room temperature and data was recorded after achieving a pre-load of 10 mN. To calculate the apparent tensile modulus, the manually determined linear portion of the stress/strain curve was used.

AFM microindentation: AFM microindentation experiments were performed in PBS on a NanoWizard^®^ ULTRA Speed AFM system (JPK Instruments AG, Berlin) equipped with an inverted optical microscope (Axio Observer.D1, ZEISS) 1 day after casting. Colloidal probe preparation and characterization by imaging a calibration grating (TGT1, NT-DMT Spectrum Instruments) and microindentation force tests were done as described in Kain et al. (Kain et al., 2018). Force volume maps were recorded at 2 μm/s displacement speed with an applied load of 2 nN. The maps were done in an evenly spaced 5-by-5 grid covering an area of 20 μm × 20 μm. For each hydrogel of different fibrinogen concentration (7.5, 10, 20 and 30 mg/ml), two samples of different fabrication batches were tested with three force volume maps per sample from different locations. This totals to a maximum of 75 force curves per sample, 150 per fibrinogen concentration category, barring bad quality curves that have been disregarded during data analysis. For data analysis, force versus indentation data were analyzed using the Oliver-Pharr method adapted for AFM microindentation tests according to (Kain et al., 2018) and (Andriotis et al., 2014). Since the elastic modulus of colloidal probes (tenths of GPa) is five to six orders of magnitude larger than the fibrin hydrogels (tenths of kPa), the apparent indentation modulus $E_{S}$ of the sample can be approximated as:

$$E_{S}=\frac{\sqrt{\pi }}{2\beta }\left(1-\nu_{S}^{2}\right)\frac{S}{\sqrt{A_{C}\left(h_{C}\right)}}$$

$$h_{C}=h_{max}-\varepsilon \frac{F_{max}}{S}$$

Where β=1.0226 and ε=0.75 are probe-shape-dependent empirical parameters for spherical probes according to (Oliver and Pharr 1992), $\nu_{S}=0.5$ is the Poisson’s ratio for incompressible materials (high water content), $F_{max}$ is the maximum indentation force, S the contact stiffness at maximum indentation depth and $A_{C}$ the projected indentation area dependent on $h_{C}$ , the indentation depth adjusted for deformation of the surface.

### DNA Quantification

At D0, D2 and D4 of culture, cells were harvested from 2D and 3D fibrin hydrogels by digestion in 100 µl nattokinase (Japan Bioscience Lab, CA, United States) at a concentration of 100 U/ml in PBS, pH 7.4 supplemented with 15 mmol/L ethylenediaminetetraacetic acid (EDTA) for 1 h at 37°C. To facilitate digestion, samples were constantly agitated at 700 RPM and triturated every 10 min. Afterwards, the cells were washed with 0.5 ml PBS once and the cell pellet was resuspended in 1 ml DMEM-HG without any supplements containing 1 μl Hoechst 33342 (Invitrogen, CA, United States) (5 mg/ml) yielding a final concentration of 5 μg/ml. After 1 h of incubation at 37°C in the dark, the cell suspension was resuspended and 200 μl per sample were measured in quadruplicates in black 96-well plates using the GloMax-Multi Detection System (Promega, Madison, United States) at 410–460 nm. Quantified fluorescence signals were normalized to D0 to demonstrate increase of DNA over time.

### Quantitative Reverse Transcription Polymerase Chain Reaction (RT-qPCR)

On D0, D1, D2 and D5 of differentiation, cells were harvested for RNA isolation and subsequent RT-qPCR. Cell retrieval was performed by digestion with nattokinase (as described above). RNA isolation was performed with the peqGOLD total RNA Kit (VWR International GmbH, Erlangen, Germany), reverse transcription into cDNA with the EasyScript PlusTM Reverse Transcriptase cDNA Synthesis Kit (ABM, Richmond, Canada) according to the manufacturers’ protocols. Transcription of 1 µg RNA was performed for 50 min at 42°C, followed by an inactivation step at 85°C for 5 min.

Quantitative PCR was performed with the KAPA Fast SYBR Fast Universal Kit (VWR International GmbH, Erlangen, Germany) in a Stratagene Mx3005P cycler (Agilent Technologies, Santa Clara, United States). Assays were performed in triplicates with 10 ng input cDNA per reaction. Thermal cycle conditions were 5 min at 95°C, followed by 40 cycles of either 10 s at 95°C and 30 s at 60°C (“fast-two-step”), 30 s at 95°C and 1 min at 60°C (“normal-two-step”), or 15 s at 95°C, 30 s at 55°C and 30 s at 72°C (“fast-three-step”). Target cycle threshold (CT) values were normalized to the housekeeping gene *Ribosomal Protein Lateral Stalk Subunit P0* (*RPLP0*) [known to be stably expressed independent of mechanical stress (Bayer et al., 2014)] and compared to D0 values, as well as to the corresponding 8 kPa value of the respective group using the comparative CT (ΔΔCT) method. Primer sequences, primer concentrations and used thermal profiles are listed in Table 2. Two primers for different myosin heavy chain genes were used. The primer referred to as “*MHC I*” is specific for the gene *Myh7* that encodes for slow-twitch myosin heavy chain found in type I fibers. The primer referred to as “*MHC II*” is specific for genes that encode for all different fast-twitch myosin heavy chains found in type II fibers (*i.e. Myh1*, *Myh2 and Myh4*).

**TABLE 2:** Primer sequences, primer concentrations and thermal profiles used for qPCR.

Target	Species	Primer forward	Primer reverse	Primer conc. (nM)	Thermal profile
*RPLP0*	Mouse	5ʹ-CTC​CAA​CAG​AGC​AGC​AGA-3ʹ	5ʹ-ATA​GCC​TTG​CGC​ATC​TGG​T-3ʹ	200	fast-2-step
*CCND1*	Mouse	5ʹ-TCA​AGT​GTG​ACC​CGG​ACT​G-3ʹ	5ʹ-ATG​TCC​ACA​TCT​CGC​ACG​TC-3ʹ	200	fast-2-step
*p21*	Mouse	5ʹ-CCG​TGG​ACA​GTG​AGC​AGT​TG-3ʹ	5ʹ-TGG​GCA​CTT​CAG​GGT​TTT​CT-3ʹ	200	fast-3-step
*Myf5*	Mouse	5ʹ-TGA​CGG​CAT​GCC​TGA​ATG​TA-3ʹ	5ʹ-GCT​CGG​ATG​GCT​CTG​TAG​AC-3ʹ	200	fast-2-step
*MyoD*	Mouse	5ʹ-ACT​ACA​GTG​GCG​ACT​CAG​AT-3ʹ	5ʹ-CCG​CTG​TAA​TCC​ATC​ATG​CC-3ʹ	200	normal-2-step
*MyoG*	Mouse	5ʹ-GGT​CCC​AAC​CCA​GGA​GAT​CAT-3ʹ	5ʹ-ACG​TAA​GGG​AGT​GCA​GAT​TG-3ʹ	200	normal-2-step
*Tnnt I*	Mouse	5ʹ-AAA​CCC​AGC​CGT​CCT​GTG-3ʹ	5ʹ-CCT​CCT​CCT​TTT​TCC​GCT​GT-3ʹ	200	fast-2-step
*MHC I*	Mouse	5ʹ-CTC​AAG​CTG​CTC​AGC​AAT​CTA​TTT-3ʹ	5ʹ-GGA​GCG​CAA​GTT​TGT​CAT​AAG​T-3ʹ	200	fast-3-step
*MHC II (all isoforms)*	Mouse	5ʹ-GAG​GGA​CAG​TTC​ATC​GAT​AGC​AA-3ʹ	5ʹ-GGG​CCA​ACT​TGT​CAT​CTC​TCA​T-3ʹ	200	fast-3-step
*RPLP0*	Human	5ʹ-GAA​ATC​CTG​AGT​GAT​GTG​CAG​C-3ʹ	5ʹ-TCG​AAC​ACC​TGC​TGG​ATG​AC-3ʹ	200	normal-2-step
*CCND1*	Human	5ʹ-GTG​CCA​CAG​ATG​TGA​AGT​TCA​TT-3ʹ	5ʹ-CTC​TGG​AGA​GGA​AGC​GTG​TG-3ʹ	200	fast-2-step
*p21*	Human	5ʹ-TGG​AGA​CTC​TCA​GGG​TCG​AAA-3ʹ	5ʹ-GGC​GTT​TGG​AGT​GGT​AGA​AAT-3ʹ	200	fast-3-step
*Myf5*	Human	5ʹ-ATG​CCA​TCC​GCT​ACA​TCG​AG-3ʹ	5ʹ-ATT​CGG​GCA​TGC​CAT​CAG​AG-3ʹ	400	fast-2-step
*MyoD*	Human	5ʹ-CAC​GTC​GAG​CAA​TCC​AAA​CC-3ʹ	5ʹ-TGT​AGT​CCA​TCA​TGC​CGT​CG-3ʹ	400	normal-2-step
*MyoG*	Human	5ʹ-CAT​CCA​GTA​CAT​CGA​GCG​CC-3ʹ	5ʹ-GCA​GAT​GAT​CCC​CTG​GGT​TGG-3ʹ	200	fast-2-step
*Tnnt1*	Human	5ʹ-ACC​TGG​TCA​AGG​CAG​AAC​AG-3ʹ	5ʹ-CAG​GAG​GGC​TGT​GAT​GGA​G-3ʹ	200	fast-3-step
*MHC I*	Human	5ʹ-ACA​CAC​TTG​AGT​AGC​CCA​GG-3ʹ	5ʹ-ACG​GTC​ACT​GTC​TTG​CCA​TA-3ʹ	400	normal-2-step
*MHC II (all isoforms)*	Human	5ʹ-TAC​TGC​ACA​CCC​AGA​ACA​CC-3ʹ	5ʹ-TTT​TCT​TCT​GCA​TTG​CGG​GC-3ʹ	200	normal-2-step

### Immunofluorescence Staining and Analysis

On day 5 of differentiation, cells were fixed with 4% paraformaldehyde (PFA; Roth, Karlsruhe, Germany) overnight at 4°C, washed with distilled water and permeabilized with Tris-buffered Saline/0.1% Triton X-100 (TBS/T) (v/v) for 15 min at room temperature. Blocking was performed with PBS/T-1% bovine serum albumin (BSA) (w/v) at room temperature for 1 h. The primary antibody targeting all MHC isoforms (MF 20, Developmental Studies Hybridoma Bank, Iowa, United States, RRID: AB_2147781) was diluted 1:300 in PBS/T-1% BSA (w/v) and incubated overnight at 4°C. Subsequently, the hydrogels were washed with PBS/T and incubated with the secondary antibody labelled with Alexa Fluor 488 (Life Technologies, Lofer, Austria) diluted 1:400 in PBS/T-1% BSA at 37°C for 1 h. Nuclei were by staining with 4′,6-diamidino-2-phenylindole (DAPI) diluted 1:1000 in PBS/T-1% BSA for 10 min at room temperature. All stainings were analyzed with a Leica DMI 6000b inverted microscope (Leica Microsystems GmbH, Wetzlar, Germany).

For assessment of myotube maturation, fusion index and myotube alignment of MHC-stained samples were analyzed using the imaging analysis software Fiji. Fusion index was calculated as the ratio of nuclei in fused myotubes (defined as myotubes with a minimum of three nuclei) to the total number of nuclei. Myotube alignment was calculated as the deviation between the axis of orientation of single myotubes from the mean axis of orientation.

### Statistical Analyses

All statistical calculations and depiction of data was performed with GraphPad Prism Software (GraphPad Software Inc., SanDiego, United States). Data is presented in box and whisker plots (minimum to maximum) or as time-lines relative to D0 (means are shown), except Figure 5C that is presented as mean + standard deviation. All data sets were analyzed for normal distribution with the D’Agostino and Pearson omnibus normality test. Comparison between groups was performed with one-way ANOVA with Tukey’s multiple comparison test, Kruskal–Wallis test with Dunn’s multiple comparison test or two-way ANOVA with Sidak’s multiple comparison test, as indicated in the figure legends with *p* values ≤ 0.05 considered statistically significant.

## Results

### Stiffness of Fibrin Hydrogel Scaffolds Directly Correlates With Total Fibrinogen Content

To ensure optimal conditions to prime cells for developing into muscle-like tissue, providing the right stiffness of the biomaterial is of high relevance. Therefore, we analyzed the effect of varying fibrinogen concentrations on the elastic properties of fibrin hydrogel. Within the tested fibrinogen range (7.5 mg/ml – 30 mg/ml), an almost linear relationship between apparent tensile modulus and fibrinogen concentration was observed (Figure 1A) by tensile mechanical testing of intact ring-shaped constructs and by AFM microindentation measurements. Stiffness values from AFM microindentation measurements were significantly lower compared to those measured by test-to failure tensile measurements (Figure 1A).

### Matrix Properties Affect Proliferation of Murine and Human Myoblast Differently

**FIGURE 2:**
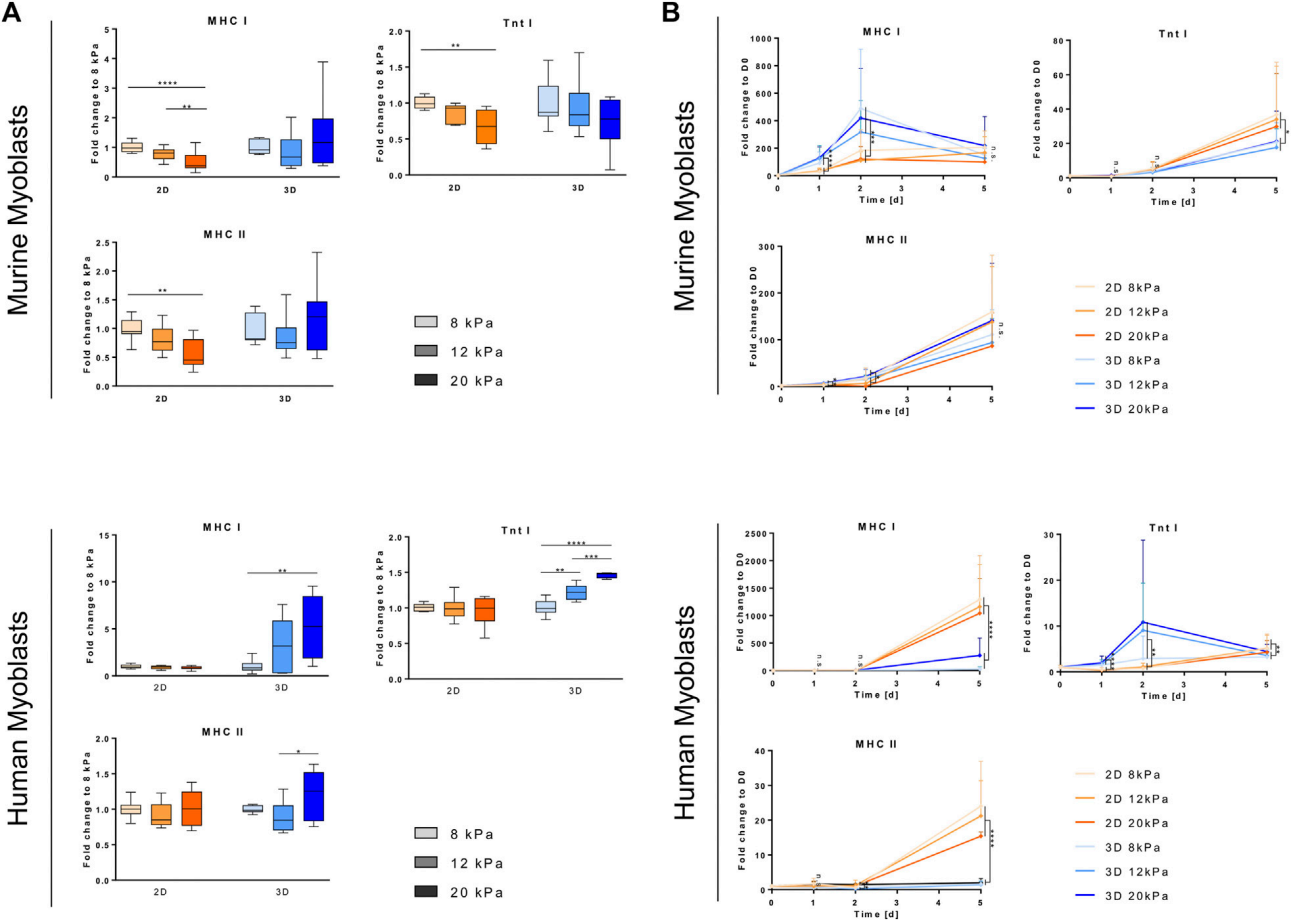
Substrate stiffness and architecture affect myoblast proliferation. Murine and human myoblasts were cultured on fibrin-coated cell culture plates (2D) or encapsulated in fibrin hydrogels (3D) with different fibrinogen (Fbg) concentrations (5, 10 and 20 mg/ml) for 4 days under proliferative conditions. DNA content of samples was assessed by Hoechst staining. **(A)** DNA content of each fibrinogen concentration (5, 10 and 20 mg/ml), culture set up (2D and 3D) and cell type (murine and human myoblasts) was normalized to their respective D0 values (dotted line indicates D0 level). Data is shown as box and whiskers (min to max); *N* ≥ 2, *n* ≥ 6; one-way ANOVA with Tukey’s multiple comparison test was performed comparing all fibrinogen concentrations of each day with each other, *p < 0.05, **p < 0.01, ****p < 0.0001. **(B)** DNA content of all fibrinogen concentrations (5, 10 and 20 mg/ml) of each culture set up (2D and 3D) and cell type (murine and human myoblasts) was normalized to their respective D0 values. Data is shown as mean ± SD; *N* ≥ 2, *n* ≥ 6; two-way ANOVA with Sidak’s multiple comparison test was performed comparing culture set ups (2D *vs*, 3D) of each cell type (* indicates murine myoblasts, # indicates human myoblasts) with each other, ****/^####^ *p* < 0.0001.

Cell cycle progression is one of the cellular processes known to be largely affected by material properties. One of our aims was thus to study the differential effects of substrate stiffness and geometry on cell proliferation. Human and murine myoblasts were cultured either on fibrin-coated well plates (“2D”) or embedded in fibrin hydrogels (“3D”). Fibrinogen concentrations of 5, 10 and 20 mg/ml were chosen as they result in apparent tensile moduli in a range relevant for SMTE (5.1, 10.3 and 20.6 kPa, respectively) (Engler et al., 2004). Results of DNA quantification clearly suggest that stiffness, as well as culture type influenced cellular proliferation. When cultured in 2D, increased stiffness resulted in increased proliferation with significant differences in human, but not in murine myoblasts (Figure 2A, left panels). By D4, DNA amounts increased on average 6.4-, 7.1-, and 7.9-fold in murine myoblasts and 5.8-, 6.0-, and 6.6-fold in human myoblasts (compared to D0) when cultured on fibrin hydrogels with ascending apparent elastic moduli. Upon incorporation into fibrin hydrogels, however, the opposite effect was seen with significant differences between the fibrinogen concentrations in both cell lines (Figure 2A, right panels). In this setup, DNA amounts increased on average 3.1-, 2.8-, and 1.6-fold in murine myoblasts and 4.0-, 3.6-, and 2.6-fold in human myoblasts (compared to D0) in fibrin hydrogels with fibrinogen concentrations of 5, 10 and 20 mg/ml, respectively. Comparing changes over time, culture in 2D led to a significantly higher increase in DNA content than in 3D in both cell lines and all scaffold rigidities (Figure 2B). Interestingly, the difference between 2D and 3D culture was less pronounced in the softest hydrogel (5 mg/ml fibrinogen), in comparison to the more rigid ones. Furthermore, it is notable that the DNA content of murine myoblasts increased less after D2 of culture than in human myoblasts, particularly in softer hydrogels (Figure 2B).

### Scaffold Geometry Affects Early Myogenesis in Murine and Human Myoblasts

To gain insight into the effects of material geometry and mechanical properties on myogenesis in human and murine myoblasts, we changed the experimental setup to promote differentiation rather than proliferation. Myoblasts were seeded at a high density (1 × 10^5^ cells/cm^2^), which is known to promote onset of differentiation (Buckingham 2002) and treated with their respective differentiation media 1 day after transferring them into/onto the biomaterial. Furthermore, fibrinogen concentrations were adapted to reflect rigidities relevant for SMTE. In accordance with Engler *et al.* (Engler et al., 2006), who defined a range between 8 and 17 kPa as suitable for myogenic specification in 2D, and taking into account the fact that human muscle is characterized by higher rigidities (*see* Table 1), we generated 2D and 3D fibrin clots with apparent tensile moduli of 8, 12 and 20 kPa (which corresponds to fibrinogen concentrations of 7.8 mg/ml fibrinogen, 11.67 mg/ml fibrinogen and 19.42 mg/ml fibrinogen). Calculation of fibrinogen concentrations for respective apparent tensile moduli was based on the results gained from tensile testing (Figure 1A). To observe the progression of myogenesis in different culture setups, we analyzed the gene expression of a set of early- (*Myf5, MyoD*) and mid-stage (*MyoG*) myogenic markers, as well as a marker for proliferation/active cell cycle (*CCND1, p21*) with RT-qPCR.

**FIGURE 3:**
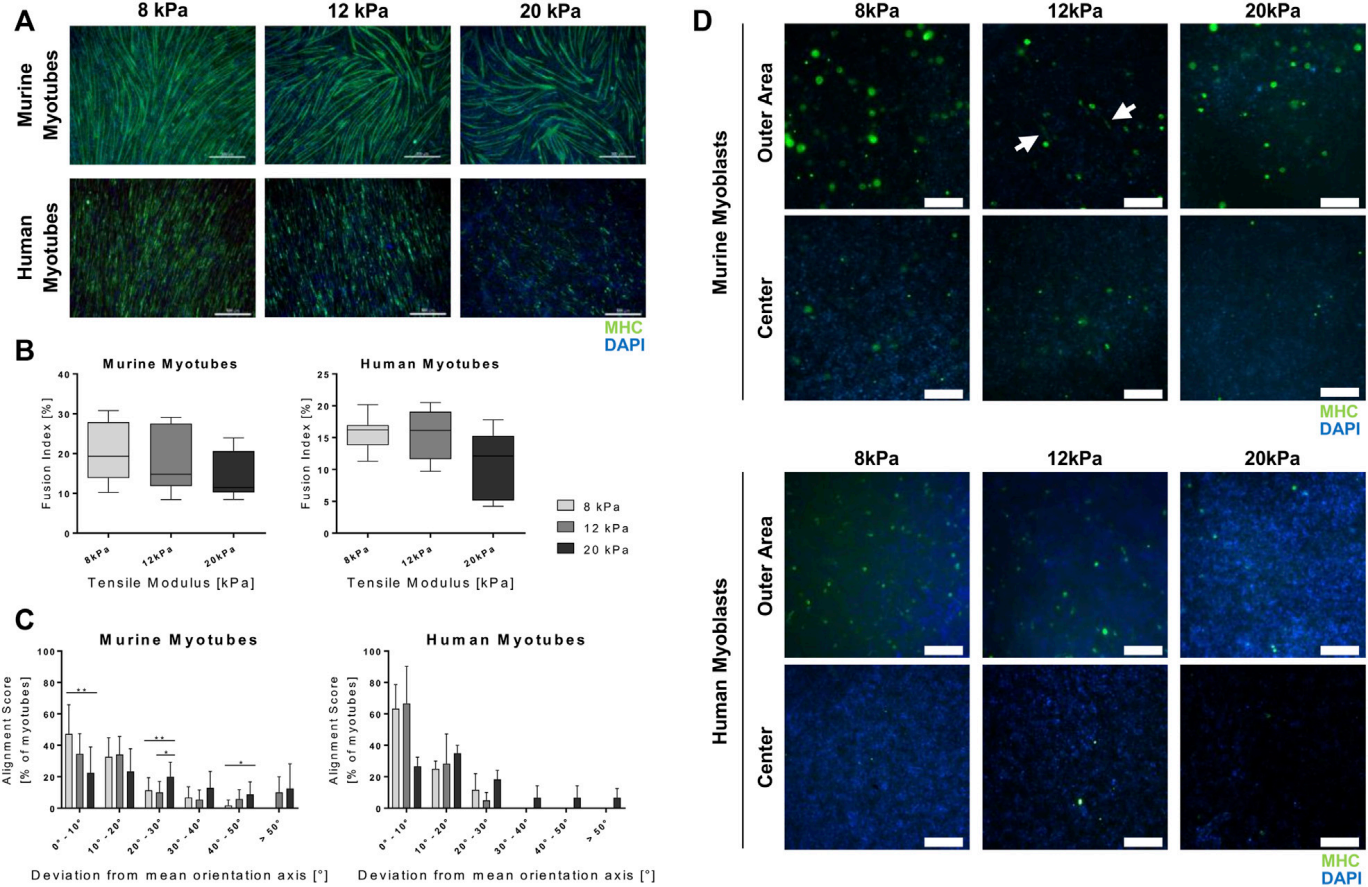
Culture in a 3D environment slows down progression of myogenesis in myoblast cell lines. Murine and human myoblasts were differentiated on fibrin-coated cell culture plates (2D) or encapsulated in fibrin hydrogels (3D) with different stiffness (apparent tensile modulus of 8, 12 and 20 kPa) for 5 days. They were seeded in growth medium that was replaced by differentiation medium on D1. mRNA expression of marker genes specific for early, mid and late stage myogenesis [*Myf5* **(A)**, *MyoD* **(B)** and *myogenin* (*MyoG*) **(C)**, respectively] and cell cycle progression [*cyclin D 1* (*CCND1*) **(D)** and *p21* **(E)**] was assessed by RT-qPCR. Fold change expression levels were normalized to D0 control samples of each cell line and relative expression over time is shown as a linear curve between means of fold-change values **(A**–**D)** or normalized to the 8 kPa group for each condition on day 5 (D5) **(E)**; *N* = 3, *n* ≥ 8; two-way ANOVA with Sidak’s multiple comparison test was performed comparing all rigidities (8, 12 and 20 kPa) of the two (culture set ups (2D *vs* 3D) with each other **(A**–**D)** or one-way ANOVAq with Tukey’s multiple comparison test was performed comparing all rigidities for each condition (2D and 3D) with each other **(E)**, no significant difference (n.s.), *p < 0.05, **p < 0.01, ***p < 0.001, ****p < 0.0001.

Cyclin D1, encoded by the *CCND1* gene, is a member of the highly conserved cyclin family, which, while being expressed in all adult tissue, is subjected to periodic fluctuations in its abundance throughout the cell cycle. Cyclin D1 regulates the activity of the cyclin dependent kinases four and six that are required for transition from the G1 to the S-phase (Sherr and Roberts 1999). One day after transferring myoblasts from cell culture plastic to different biomaterial settings, these fluctuations in *CCND1* transcription depending on cell cycle state became apparent. For both cell lines, embedding into a 3D fibrin matrix, independent of rigidity, led to a drastic drop in *CCND1* gene expression compared to D0 (average 4.7- and 2.8-fold downregulation, respectively) despite culture in GM. Culture on top of fibrin-coated wells, on the other hand, induced significantly higher *CCND1* expression than in the 3D setup, with only a slight decrease in murine and even an average 5.1-fold increase in human myoblasts (Figure 3D). During the course of the experiment, *CCND1* gene expression levels in all cultured murine myoblasts converged to similar levels of an average 2.4-fold decrease compared to D0 (Figure 3D, left panel), whereas it dropped an order of magnitude lower, to less than 0.1 times the expression of D0 in human myoblasts (Figure 3D, right panel). While in all other setups the different material rigidities had no effect on *CCND1* expression levels, culture on the stiffest matrix (20 kPa) did alter its expression rate in human myoblasts. They showed comparatively low fold-change increases 24 h post-plating (3.2-fold increase in the 20 kPa group *vs* an average 6-fold increase in the others) and a peak of a 6.3-fold increase on D2 (Figure 3D, right panel). *p21* gene expression was assessed on D5 as an indicator of cell cycle exit (Zhang et al., 1999). An up to 1.8-fold downregulation of *p21* transcription was observed in murine myoblast upon culture in or on gels with higher apparent elasticities. In human myoblasts, however, there was a non-significant trend of higher apparent elasticities leading to increased mean values of *p21* transcription (Figure 3E).

The major regulators of the onset of myogenesis are a set of transcriptional regulators, the myogenic regulatory factors (MRFs). The MRFs observed in this study, *Myf5*, *MyoD* and *MyoG*, are basic helix loop helix transcription factors that are themselves expressed in hierarchical time-dependent manner and their expression can be used to characterize the differentiation state of myogenic cells (Bentzinger, Wang, and Rudnicki 2012; S.; Yokoyama and Asahara 2011). *Myf5* and *MyoD* are required for myoblast determination from the satellite cells state. *Myf5* expression peaks before and during early phases of differentiation and diminishes completely afterwards; *MyoD* peaks in early differentiation, but is constantly expressed at low levels throughout terminal differentiation (Rudnicki et al., 1993). Expression of *MyoG* during differentiation is dependent on prior *MyoD* expression and is required for terminal differentiation (Londhe and Davie 2011; S.; Yokoyama and Asahara 2011). Overall, this time-dependent cascade-like expression pattern was observed in both cell lines, and in all different matrix setups with some alterations in the extent and timing of upregulation of the different transcription factors. Upregulation of *Myf5* was observed 24 h into differentiation in all setups, except when murine myoblasts were cultured on a 2D fibrin matrix (Figure 3A). In this condition, transcriptional levels of *Myf5* remained constant at all time points throughout the observed culture period (Figure 3A, right panel). The expression levels on D2 compared to D0, however, differed between the two species. A moderate (average 1.8-fold) upregulation was observed in murine myoblasts in the 3D setup, whereas increases in the range from 17.2- to 208-fold upregulation were measured in human myoblasts. Upregulation was observed in a similar range within the different groups independent of substrate rigidity, except for one group, human myoblasts grown on the most rigid substrate (20 kPa). Those showed a more pronounced upregulation on D2 (208-fold increase *vs* an average of 17.3-fold increase; Figure 3A, right panel). In murine myoblasts, *Myf5* expression remained at similar levels throughout the observed culture time with a significantly higher gene expression in the 3D compared to the 2D setup (Figure 3A, left panel). In human myoblasts, on the other hand, transcription of *Myf5* dropped drastically by D5 to expression between 0.24- to 0.31-fold the D0 levels independent of biomaterial stiffness and architecture (Figure 3A, right panel). In all studied groups, the average *MyoD* transcriptional levels were higher in the 2D setup compared to the 3D setup. Murine myoblasts differentiated on fibrin showed significantly higher *MyoD* expression levels compared to 3D-cultured counterparts, in which even downregulation compared to D0 was measured on the first 2 days of differentiation (Figure 3B, left panel). *MyoD* levels in human myoblasts did not exceed the basal level prior to transfer to the biomaterial at any of the observed time points with a sharp drop on D1 of an average 10.8-fold decrease. When cultured in a 2D setup, however, expression levels reached the basal levels of *MyoD* expression by the second day of differentiation, but not in the 3D setup (Figure 3B, right panel). The trend concerning differences between substrates of different stiffness was also confirmed in *MyoD* expression: overall, substrate stiffness within the myogenic range did not impact changes in expression levels, except for human myoblasts grown in the most rigid substrate (20 kPa), which showed a downregulation by the end of the differentiation period (0.25-fold change *vs* an average of 1.4-fold increase) (Figure 3B, right panel). *MyoG* expression increased by the second day of differentiation in all treatment groups. In murine myoblasts, it further proceeded to an average 29-fold increase by D5, independent of scaffold type and stiffness (Figure 3C, left panel). Human myoblasts cultured embedded in 3D fibrin matrices showed comparably moderate fold changes of 126.8 on average, while culture of these cells on top of biomaterials induced a significantly higher upregulation with fold change increases of 1,724 on average compared to D0 (Figure 3C, right panel).

### Matrix Properties Have Different Effects on Terminal Differentiation in Murine and Human Myogenic Cells

**FIGURE 4:**
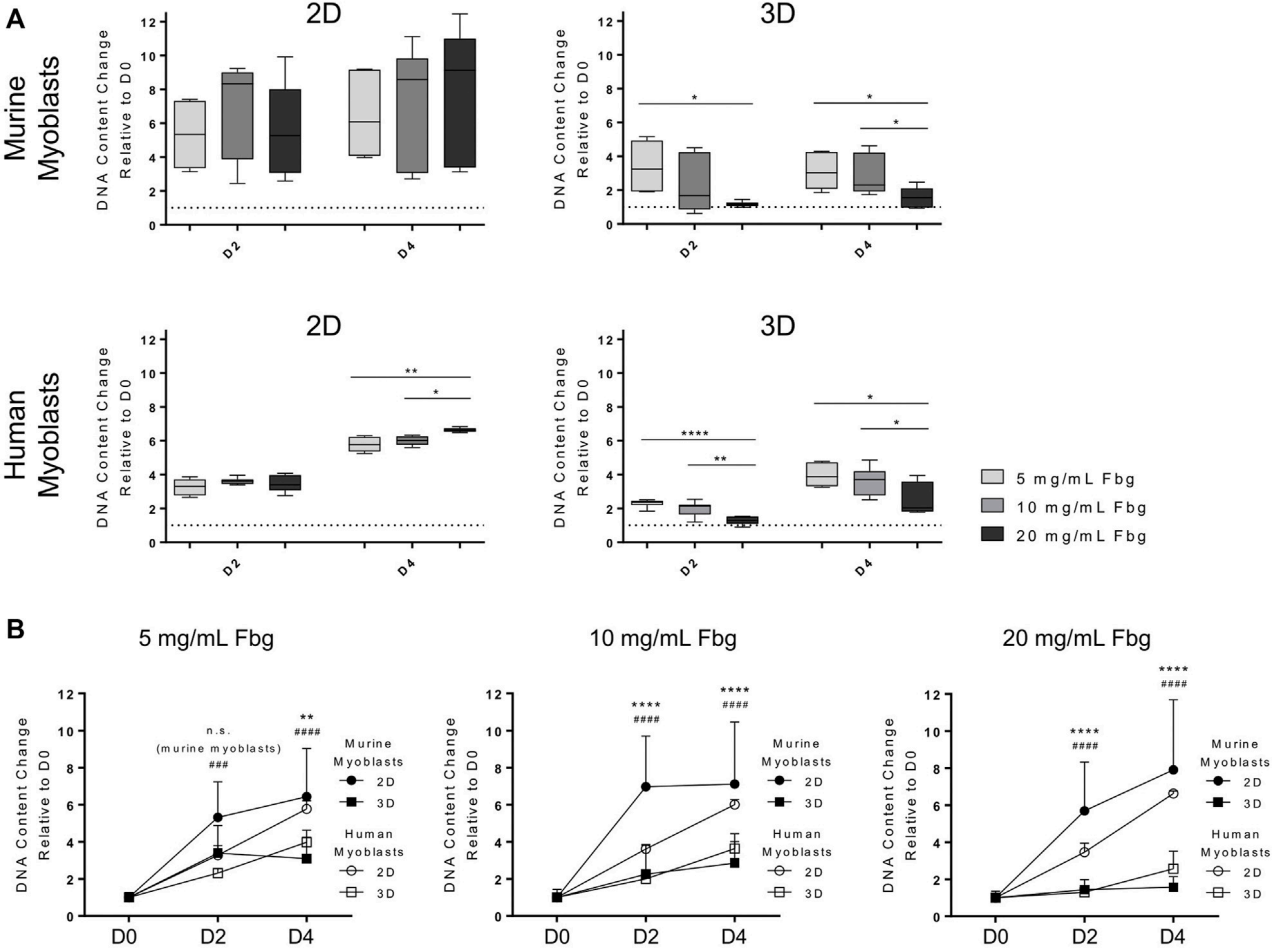
Culture in a 3D setup affects impact of substrate stiffness and myogenic outcome. Murine and human myoblasts were differentiated on fibrin-coated cell culture plates (2D) or encapsulated in fibrin hydrogels (3D) with different stiffness (apparent tensile modulus of 8, 12 and 20 kPa) for 5 days. mRNA expression of marker genes for terminal differentiation (*troponin T I* (*Tnnt I*), *myosin heavy chain I* (*MHC I*) and *myosin heavy chain II* (*MHC II*) was assessed by RT-qPCR. **(A)** Fold change expression levels on day 5 (D5) were normalized to the 8 kPa group for each condition. Data is shown as box and whiskers (min to max); *N* = 3, *n* ≥ 8; one-way ANOVA with Tukey’s multiple comparison test was performed comparing all rigidities for each condition (2D and 3D) with each other, *p < 0.05, **p < 0.01, ***p < 0.001, ****p < 0.0001. **(B)** Fold change expression levels were normalized to D0 control samples of each cell line and relative expression over time is shown as a linear curve between means of fold-change values; *N* = 3, *n* ≥ 7 two-way ANOVA with Sidak’s multiple comparison test was performed comparing all rigidities (8, 12 and 20 kPa) of the two (culture set ups (2D *vs* 3D) with each other; no significant difference (n.s.), *p < 0.05, **p < 0.01, ****p < 0.0001.

Terminal myogenic differentiation is accompanied by the development of a functional muscular ultrastructure. Successful development of said structure is dependent on production and arrangement of structural and motor proteins (Lieber and Jan, 2000). Troponin T and myosin are proteins required for skeletal muscle contraction, thus its functionality. The troponin subtype T1, encoded by the gene *Tnnt I*, is a part of the troponin complex and therefore required for Ca^2+^-induced striated muscle contraction (Wei and Jin 2011). Myosin is the most prominent protein required for muscle contraction and consists of several subunits. Its heavy chain [myosin heavy chain (MHC)] appears in different isoforms, whose expression transitions during development and throughout *in vitro* differentiation in a distinct temporal pattern. The isotypes most prominent in slow muscle fibers (MHC I) occur prior to those specific to fast fibers (MHC IIa, MHC IIx, MHC IIb) (Brown, Parr, and Brameld 2012). Analyzing the expression of the genes encoding for troponin T 1, MHC I and all MHC II isoforms (*Tnnt I, MHC I and MHC II*), it was apparent that culture in a 2D compared to a 3D setup affected terminal differentiation drastically. Furthermore, the influences of scaffold architecture differed in the two cell lines. In murine myoblasts grown in the 2D setup, transcriptional levels of all observed markers significantly decreased with increasing substrate stiffness to an average two-fold downregulation comparing cells grown in the softest (8 kPa) to the stiffest (20 kPa) material (Figure 4A, upper panels). On the other hand, under these conditions, substrate stiffness did not alter gene expression of *Tnnt I, MHC I* and *MHC II* in human cells (Figure 4A, lower panels). Interestingly, the exact opposite effects were observed in the 3D setup. When incorporated into fibrin hydrogels, terminal differentiation was not affected by different substrate rigidities in murine myoblasts (Figure 4A, upper panels). In human myoblasts, in contrast, incorporation in stiffer hydrogels led to an average 3.3-fold upregulation of the expression of the aforementioned structural genes (Figure 4A, lower panels). Observing the time course, it was evident that human cells were more susceptible to influence by incorporation into 3D hydrogels in terms of terminal differentiation. In murine myoblasts, upregulation of transcriptional levels of late-stage myogenesis marker genes are on a similar level in cells cultured in 2D and 3D. Here, the only variation between the setups was observed in gene expression of *MHC I* that was significantly higher in 3D-cultured cells than their 2D counterpart 1 day post induction of differentiation (Figure 4B, upper panels). Notably, the differences between 2D and 3D culture were more pronounced in human cells. An average 14-fold higher upregulation of *Tnnt I* was measured 1 day after induction of differentiation. This effect, however, was not evident by the end of the culture period. In contrast, the expression of the two MHC isoforms, *MHC I* and *MHC II*, at the end of the observed period was on average 11-fold higher in 2D than in 3D environments (Figure 4B, lower panels).

### Matrix Stiffness Affects Morphology and Patterns of Myotube Arrangement

**FIGURE 5:**
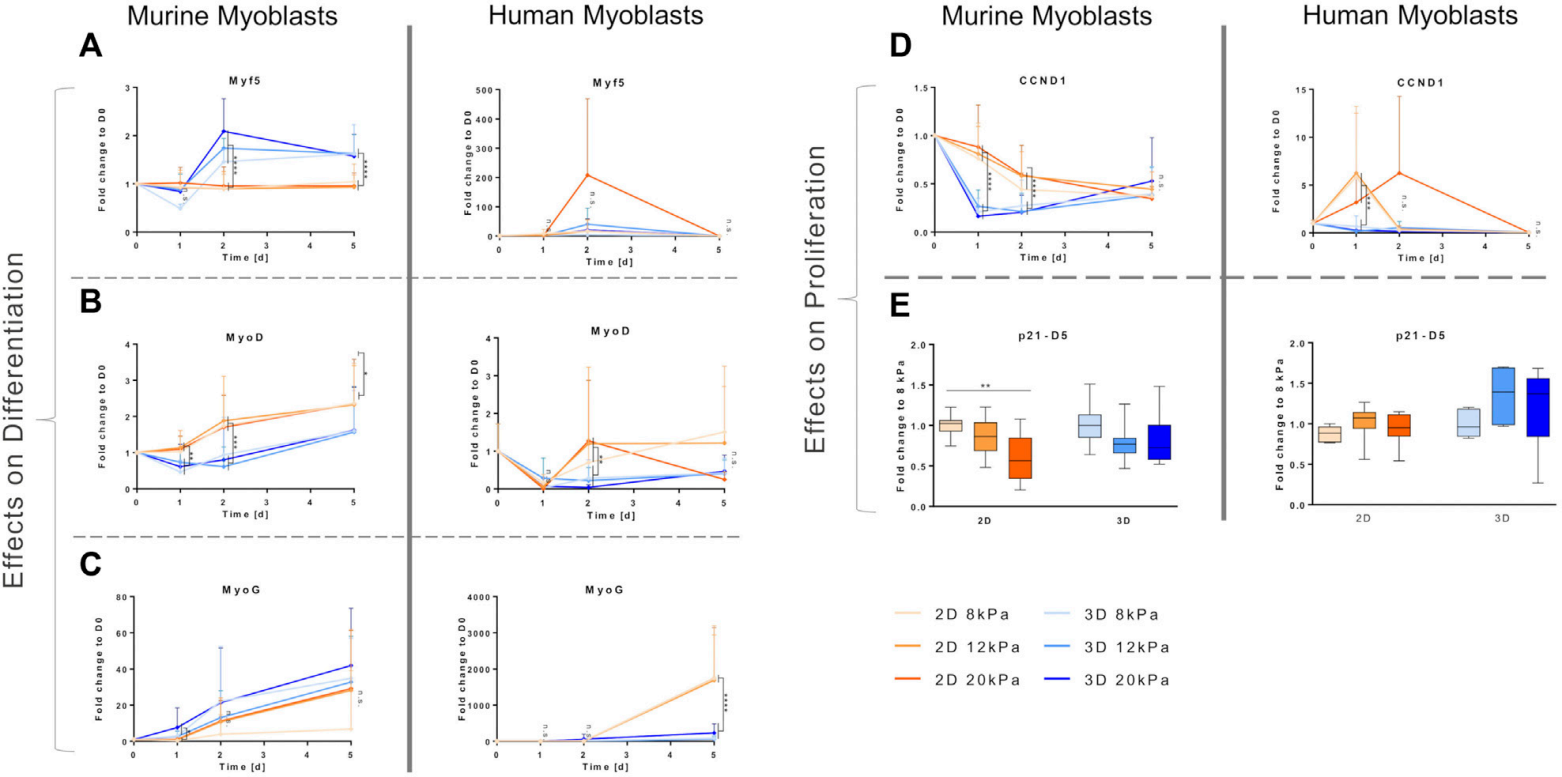
Culture on more rigid substrates interferes with myotube maturation. Murine and human myoblasts were differentiated on fibrin-coated cell culture plates (2D; **(A**–**C)** or embedded in fibrin hydrogels (3D; **(D)** with different stiffness (apparent tensile modulus of 8, 12 and 20 kPa) for 5 days. **(A)** Representative images of immunofluorescence (IF) stainings for MHC (green) with nuclei stained with DAPI (blue). Scale bars represent 500 µm. **(B)** Myotube fusion index of IF stainings (MHC and DAPI); analyzed as the ratio of fused nuclei to total nuclei per visual field (*N* = 4, *n* ≥ 10 for murine myoblasts, *N* = 3, *n* ≥ 9 for human myoblasts with four regions of interest, at least 400 nuclei per sample and 4000 nuclei per experimental group analyzed. Data is shown as box and whiskers (min to max); one-way ANOVA with Tukey’s multiple comparison test was performed comparing all rigidities with each other with no significant differences found. **(C)** Myotube alignment score of IF stainings (MHC and DAPI); analyzed as the deviation of single myotubes from the main axis of orientation, represented as a histogram of the percentage of myotubes deviating in the respective interval of 10° angles. (*N* = 4, *n* ≥ 10 for murine myoblasts, *N* = 3, *n* ≥ 9 for human myoblasts with at least 180 myotubes per experimental group analyzed. Data is shown as mean + SD; one-way ANOVA with Tukey’s multiple comparison test was performed comparing all rigidities of each deviation interval, *p < 0.05, **p < 0.01. **(D)** Representative images of IF stainings for MHC (green) with nuclei stained with DAPI (blue) taken either at the edge or in the center of the hydrogels. White arrows indicate myocytes. Scale bars represent 200 μm; *N* = 2, *n* = 4.

A key aspect of terminal myogenic differentiation is the arrangement of myotubes in a functional way. Thus, we analyzed the effect of substrate stiffness on myotube morphology, particularly alignment and fusion. Myoblasts were cultured on fibrin-coated well-plates and differentiated for 5 days prior to immunofluorescence staining of MHC, which confirmed the findings of the gene expression analysis of late-stage myogenic markers. When differentiated on stiffer substrates, myotubes derived from both myoblast lines showed lower MHC protein expression (Figure 5A), which is in accordance with the corresponding qPCR data (Figure 4A). Quantifications of myotube fusion and alignment showed the same trend of decreased myotube maturation with increasing substrate stiffness. The fusion indices of cells grown on more rigid materials were lower than those grown on softer ones. The ratios of fused nuclei to total number of nuclei were 20.3, 18.5 and 14.8% in murine and 15.8, 15.4 and 11.0% in human myotubes grown on 8, 12 and 20 kPa substrates, respectively (Figure 5). Myotube alignment was also negatively impacted by culture on stiffer substrates. When grown on the softest substrate (8 kPa), the majority of myotubes showed a 0°–10° deviation from the main axis of orientation in both species. Culture on the most rigid substrate (20 kPa), on the other hand, led to deviations larger than 20° from the main axis (Figure 5C). Interestingly, human myoblasts differentiated less than murine ones, in terms of MHC expression and myotube fusion (Figures 5A,B). The detrimental effect of culture on stiffer substrates on cell alignment, however, was less severe than in murine cells (Figures 5A,C). Furthermore, images of IF stainings of myotubes differentiated embedded in 3D fibrin hydrogels were taken at different sites of the hydrogel. Evidently, more cells were located at the outer area, whereas almost no cells were found in the centers of the hydrogels. Moreover, differentiation to myocytes only took place in the outer areas, and not in the center (Figure 5D).

## Discussion

The aim of this study was to examine the effect of different scaffold geometries (2D *vs* 3D culture) and material stiffness on the behavior of myoblasts. Two different myoblasts lines were compared, murine C2C12 cells and human C25 cells. We chose the C2C12 cell line as a basis for this study, since SMTE strategies are often developed and characterized using cell lines. The murine cell line C2C12 is used most frequently due to its easy handling and cost. Moreover, its behavior is well-studied and predictable. Aiming at increased relevance in terms of translatability to humans, human-derived cell lines, such as the C25 line, can be employed (Maleiner et al., 2018). Frequently, applying methodology established in murine lines fails when using human cells due to the murine cells’ divergent behavior. To ensure that there are no substantial differences between the two cell lines, we characterized their differentiation behavior on cell culture plastic as a preliminary experiment (Supplementary Figure S1). Another potential pitfall in the development of TE strategies is that they often rely on 3D cell carrier systems for the creation of tissue constructs. In this regard, one must keep in mind that knowledge gained on cellular behavior in 2D cultures cannot be translated to 3D environments. Given that *in vivo* cells grow in complex structures, it is obvious that 2D setups do not account for the intricate spatial arrangement of tissues. Few studies, however, have addressed the different impacts on cellular behavior triggered by 2D culture (Birgersdotter, Sandberg, and Ernberg 2005). To analyze the impact of 3D culture specifically, we grew cells under otherwise standardized conditions in two different setups (2D and 3D), where scaffold formulation translating to differential stiffness and network density was the only variable (Figure 1).

Furthermore, there is strong evidence that the stiffness of the substrates cells are cultured on influences their behavior in many ways. This is of great interest for TE applications, as it also affects the differentiation potential drastically. Moreover, maturity of engineered tissues as well as their engraftment efficiency and functionality when implanted *in vivo* are affected (Han, Jang, and García 2017; Serena et al., 2010; Palchesko et al., 2012; Gilbert et al., 2010; Engler et al., 2004). Data on optimal substrate stiffness for creation of mature muscle constructs differ greatly. Optimal values for myoblasts differentiation found in literature range from a Young’s modulus of 11 kPa (Engler et al., 2004) to 1.72 MPa (Palchesko et al., 2012). Frequently, it is stated that optimal substrate stiffness lies in the range of the stiffness of actual skeletal muscle tissue. As shown in Table 1, those values differ significantly as well. In the context of the apparent lack in comparability of different studies, not only the ranges of compared Young’s moduli vary. Additionally, the methods for Young’s modulus measurements differ as well, as it can be performed through tensile testing (Hu et al., 2011; Palchesko et al., 2012; Romanazzo et al., 2012) or AFM (Engler et al., 2004, 2006; Gribova et al., 2013). A review by McKee *et al.* pointed out that elastic modulus values of muscle are ∼70 times higher when assessed with tensile testing in comparison to indentation measurements (McKee et al., 2011). These variations in measured Young’s moduli can affect the comparability of different studies. As a first step to characterize the material used in this study, correlations between increasing fibrinogen concentrations of fibrin hydrogels and tensile and indentation moduli were analyzed (Figure 1). AFM microindentation measurements take place at lower length scales compared to the tensile tests performed. Therefore, AFM microindentation measurements are more sensitive to locally heterogeneous properties of the sample, for example microsized pores, roughness, and locally changing stiffness, resulting in higher SDs. The fact that our measured microindentation moduli are always lower compared to the tensile moduli from macroscale tests can be due to a number of reasons, including viscoelastic sample properties and different strain rates. Additionally, the stress states are different and more complex in indentation-type loading compared to tensile or compressive tests. Indentation results in compressive, tensile, and shear stresses all taking place at the vicinity of the indentation point. As described, the models used in both the tensile and the indentation experiments are by design purely elastic models. Hydrogels are generally viscoelastic due to their high water content, resulting in material responses that vary with strain rates. As the strain rate increases, the apparent elasticity also increases (Zhao et al., 2010; Chaudhuri et al., 2016). Furthermore, the tensile tests were conducted at much higher displacement rates of 300 μm/s compared to the 2 μm/s indentation velocity of the AFM. This may well explain the difference in apparent elasticity (indentation modulus *vs* tensile elasticity) between the two testing modalities. Although differences exist between the two methods, both experiments result in the same trend of increasing apparent elasticity with concentration of fibrinogen.

Subsequently, we assessed effects of different apparent elastic moduli on human and murine myoblast embedded in or grown on fibrin hydrogels. We compared moduli in a narrow range (from ∼5 to ∼20 kPa) aiming to identify the impact of the mechanical environment on cellular behavior. The chosen range is based on the findings by Engler *et al.*, who identified an indentation modulus of 12 kPa as optimal for skeletal muscle substrates, reflecting the stiffness of native tissue (Engler et al., 2004). Our data on the effects on proliferation demonstrate that culture on gels with higher stiffness increased proliferation (Figure 2). It is difficult to establish a relation between the obtained data to what is found in literature, as most studies compare different ranges of elastic moduli. Frequently, the analyzed stiffness is different from native muscle (Table 1), such as in a study by Boonen et al., in which substrates with 3, 21 and 80 kPa were compared (Boonen et al., 2009); a study by Gilbert *et al.* that compared a gel with 12 kPa to a polymer used for cell culture (∼3 GPa) (Gilbert et al., 2010); a study by Romanazzo *et al.*, in which only gels with a tensile modulus higher than 0.9 MPa were analyzed (Romanazzo et al., 2012), or a study by Boontheekul *et al.* that compared gels with elastic moduli of 1, 13 and 45 kPa (Boontheekul et al., 2007). However, the conclusions drawn are similar to this study: while we found that in a range between 5.1 and 20.6 kPa, stiffer hydrogels result in higher cell proliferation, others state that 21 kPa are more advantageous than 3 and 80 kPa (Boonen et al., 2009), 12 kPa than 3 GPa (Gilbert et al., 2010), or 45 kPa than 13 and 1 kPa (Boontheekul et al., 2007), and that when grown on substrates more rigid than 0.9 MPa, no differences were observed (Romanazzo et al., 2012). A study by Trensz *et al.* revealed potential reasons underlying the interference of substrate stiffness with myoblast proliferation by establishing a relation between myofiber damage, myofiber stiffness and progenitor cell proliferation. It was shown that damaged myofibers have a 4-fold increased Young’s modulus as well as a 15-fold increased number of proliferative cells compared to intact ones. Culture of explanted myogenic progenitor cells on substrates with Young’s moduli representing intact (0.5 kPa) and damaged (2 kPa) myofibers also came to the result that stiffer hydrogels mimicking damaged myofibers increase proliferation (Trensz et al., 2015).

Furthermore, the fibrin matrix itself influences cellular behavior since it acts pro-proliferative. In our study, the commercially available fibrin sealant Tissucol (Baxter Deutschland GmbH, 2017) was used for production of fibrin scaffolds. As the fibrinogen component of the sealant is derived from purified human plasma, it does not only contain fibrinogen, but also small fractions of other proteins (Buchta et al., 2005; Baxter Deutschland GmbH, 2017). It is stated by the manufacturer that only approximately 90% of the plasma protein fraction are made up of fibrinogen (Baxter Deutschland GmbH, 2017). Thus, the presence of growth and wound healing factors, such as epidermal growth factor, platelet-derived growth factors, IGF, transforming growth factor β, vascular endothelial growth factor potentially contributed to increased proliferation. Besides factors influencing cellular growth and proliferation, fibrin also contains extracellular matrix proteins that enhance cell attachment, such as fibronectin (Eppley, Woodell, and Higgins 2004; Danielsen et al., 2008). Moreover, it is known that developing and mature myotubes express α5β3 integrins that specifically bind the Arg-Gly-Asp (RGD) 572–574 motif located in the α chain of fibrinogen, which leads to further cell attachment (Sinanan et al., 2008; K.; Yokoyama et al., 1999). These effects could have been further intensified as fibrinogen concentrations used in these experiments are an order of magnitude higher than those found in blood, which range between 1.5 mg/ml and 3.5 mg/ml (Oswald, Hunt, and Lazarchick 1983).

In contrast to our observations in myoblasts grown on fibrin hydrogels, embedding them in a 3D environment yielded opposite results. In this setup, proliferation was significantly decreased with increasing fibrin hydrogel stiffness (Figure 2) and drastic decrease of cell cycle regulator *CCND1* expression (Figure 3). Furthermore, myoblasts could not develop a network and spread out in stiffer hydrogels, but rather had a globular shape (Supplementary Figure S2). Most likely, this is caused by the increased density of fibrin fibers in the network of gels with higher fibrinogen concentrations (Janmey, Winer, and Weisel 2009; Heher et al., 2015). Thus, cellular motility, supply of nutrients and oxygen, as well as waste removal are reduced or impaired in stiffer hydrogels, resulting in decreased proliferation. This can be connected to changes in a plethora of intracellular mechanisms, as reviewed by Birgersdotter *et al.* (Birgersdotter, Sandberg, and Ernberg 2005). These include altered spatial arrangement of receptors (e.g. integrins) and other cytoskeletal components compared to 2D cultures, which changes regulation of signaling molecules (e.g. focal adhesion kinase or PI3K). That, in turn, suppresses cell cycle progression, for example through upregulation of p21 (Birgersdotter, Sandberg, and Ernberg 2005). The crucial impact of high biomaterial density in 3D on cellular behavior can also be seen in the experiments assessing the influence of substrate stiffness on differentiation (Figure 5D). In our setup, the effects of factors such as altered migration potential and nutrient supply cannot be separated from the influence of 3D culture *per se*. This, however, holds true for most 3D approaches. Therefore, we want to point out the importance of accounting for those added stressors when designing TE experiments. Appropriate seeding densities are of particular importance for SMTE since they are directly related to the myogenic outcome (Buckingham 2002; Martin et al., 2013). This also presents the rationale for employing different cell densities in proliferation and differentiation experiments in this study. The high cell densities required to promote myogenic development would have potentially interfered with the cells’ proliferative behavior. Therefore, lower seeding densities were chosen for those experiments. Due to the influence of the configuration of the cells in 3D in an appropriate spatial manner, it is advisable to include a resting period after embedding myoblasts in a 3D environment. Thereby to the new surrounding is enabled and sufficient cell density ensured, as employed in other studies (Okano and Matsuda 1998; Powell et al., 2002; Heher et al., 2015).

Furthermore, we observed that human myoblasts proliferated to a lower extent than murine myoblasts (Figure 2). This inter-species difference has already been reported by several other studies *in vitro* as well as *in vivo* (Rangarajan and Weinberg 2003; Chen et al., 2006; Stern-Straeter et al., 2008). It is speculated that the lower proliferation rates of human cells can be attributed to the longer lifetime of humans, which would entail ∼10^5^ more cell divisions during their whole lifetime and severely increase the risk of accumulating mutations. Thus, it is assumed that the slower proliferation rates in humans serve as a protection mechanism from increased chance of cancer development (Rangarajan and Weinberg 2003). This could be connected to another observation we made. *CCND1* expression strongly increased in C25 cells but decreased in C2C12 cells (Figure 3) when cells were transferred from cell culture plastic to fibrin-coated well plates (2D). This differential behavior could be attributed to the fact that C25 cells, with their generally lower proliferation rates, benefit from the profitable environments of the hydrogel. C2C12 cells, on the other hand, have a high basal level of cell cycle markers and furthermore initiate the onset of myogenesis more readily (*see* below). Thus, downregulation of cell cycle markers is a logical consequence. These effects, however, were only observed immediately after the change of environment. Throughout the course of differentiation, expression levels converged, indicating that those effects were overruled by advancing differentiation.

Proliferation and differentiation are mutually exclusive processes in muscle cell development. Hence, the fact that, overall, proliferation levels are lower in C2C12 cells compared to C25 cells can be interpreted as a sign of increased differentiation in the former cell type. This is reflected in the concomitant belated decrease of *CCND1* and increase of MRF gene expression in the human muscle cell line (Figure 3, right panels). Cells grown on the stiffest matrix (apparent elastic modulus of 20 kPa) stand out particularly. Compared to all other culture setups, those cells showed a delayed peak in *CCND1* expression and still expressed *Myf5* 1 day after induction of differentiation. *Myf5* is a MRF that is expressed only for a short period of time early in the process of differentiation (Bentzinger, Wang, and Rudnicki 2012) and had ceased in all other conditions at this time point. Comparing different culture setups, *i.e.* 2D *vs* 3D culture, gene expression patterns of early MRFs *Myf5* and *MyoD* confirm the observation made concerning the effect on proliferation. While 2D culture led to a rapid decrease of proliferation, differentiation was initiated earlier than in 3D, which is indicated by higher *MyoD* levels and earlier passing of the initial Myf5 peak in 2D culture. Later stages of differentiation did not seem to be impacted in the case of murine myocytes. Human myocytes, however, profited immensely from culture in 2D in contrast to 3D, as shown by the drastically higher increase in *MyoG* expression (Figure 3C). These effects are also reflected in changed expression of the cyclin-dependent kinase inhibitor *p21* that is involved in cell cycle exit during myogenesis (Zhang et al., 1999; Hawke et al., 2003). This trend continues throughout the course of myogenic differentiation. Particularly, the observation that human myogenesis is sensitive to culture in a 3D environment is substantiated. We observed severe impairment of terminal differentiation in human myotubes (Figure 5). Increase of expression levels of genes encoding for proteins required for muscle functionality, troponin T 1 and different MHC isoforms, was significantly higher in human myotubes matured in a 2D environment. In murine myotubes, however, these types of culture just showed mild beneficial effects on a transcriptional level (Figure 4B).

Onset of terminal differentiation was not only affected by the type of scaffold (2D or 3D), but also by its stiffness. Culture of murine muscle cells on substrates with an apparent elastic modulus of 8 kPa compared to substrates with 12 or 20 kPa resulted in higher expression levels *MHC I and II* and *Tnnt I* (Figure 4A), increased myotube development and alignment and improved myoblast fusion (Figure 5). The trend that culture on softer substrates promotes myogenic differentiation is in accordance with several other studies (Hu et al., 2011; Romanazzo et al., 2012; Gribova et al., 2013). However, apparent elasticities included in these studies greatly differ from our setups and range from 51 kPa up to the MPa range. Interestingly, a study by Palchesko *et al.* came to the opposite conclusion, showing that maximum myotube length was reached on polydimethylsiloxane (PDMS) substrates with a Young’s modulus of 830 kPa, whereas culture on PDMS with 5 kPa led to lowest myotube length. However, tested substrates had different rigidities than those applied in the present study, with 5, 50, 130, 830 and 1.72 MPa being tested. Furthermore, differences between the groups are only seen in early time points of the experiment (up to 5 days), whereas after 7 days of differentiation no significant difference between the groups were observed. Therefore, it is speculated that these initial results are generated by increased proliferation leading to earlier confluence, which is a major determinant of myoblast fusion (Palchesko et al., 2012). In contrast to that, the first study that pointed out the major impact of substrate stiffness on myogenic differentiation conducted by Engler *et al.* clearly stated that a Young’s modulus at the physiological range of native muscle (especially 8 and 11 kPa) is most beneficial for myogenesis (Engler et al., 2004). A follow-up study confirmed that substrate stiffness in the myogenic range (*i.e.* 8 – 17 kPa) promotes expression of the myogenic marker *MyoD* in MSCs to a higher extent when compared to much softer (1 kPa) and stiffer (34 kPa) substrates and can even regulate fate decision towards myogenic differentiation (Engler et al., 2006). The influence on myoblast differentiation is attributed to the interference of substrate stiffness with myofibrillogenesis from nascent myofibrils. They state that a compliant substrate allows developing myofibers to generate a contractile force that promotes cell alignment and fusion, which is crucial for further maturation. Culture on rigid materials leads to high contractile forces causing development of too many stress fibers and focal adhesions, which eventually inhibits organization of actin and myosin filaments that is required for myotube development. On the other hand, if substrates are too soft, cell adhesion and spreading are impaired, which prevents generation of sufficient contractile forces to induce alignment and functional maturation (Engler et al., 2004). Apparently, the range of substrate stiffness considered optimal for force generation is different for human myoblasts compared to murine ones. In the 2D setup, changing rigidities did not affect gene expression of *MHC* and *Tnnt I* (Figure 4A), while morphological changes were the same as in murine cells (Figure 5). In 3D, however, a clear trend of stiffer materials being advantageous for onset of terminal myogenic differentiation was observed (Figure 4B).

In the context of observing cellular behavior in 3D, biomaterial density and nutrient supply need to be considered. As mentioned before, increasing substrate stiffness inevitably leads to increased material density due to higher fibrinogen content. This is supported by the observation that 3D culture in the softest hydrogel, while still having a negative effect compared to 2D, is less detrimental compared to culture in stiffer hydrogels. This was observed for proliferative behavior in both species (Figure 2) and for progression of myogenesis only in the murine cell line (Figure 4A). In this matter, the cells’ ability to degrade the matrix gains relevance. It is known that C2C12 cells have high levels of plasminogen, a fibrinolytic enzyme, that are further upregulated in the process of differentiation (Muñoz-Cánoves et al., 1997; Suelves et al., 2002). The importance of adequate rates of fibrinolysis regarding myogenic outcome of TE approaches has been shown in the past (Khodabukus and Baar 2009). Prolonged culture and improved differentiation of C2C12 cells requires balanced rates of material degradation. Excessive degradation, which is the case culturing C2C12 cells in fibrin, leads to insufficient mechanical stability of the engineered constructs, while insufficient degradation hindered differentiation, potentially due to a lack of movement and cell-cell contacts (Boontheekul et al., 2007; Khodabukus and Baar 2009). In contrast to that, the pattern of plasmin activation in the C25 cell line has not been studied yet. This presents another potential key difference in the adaption of these cell lines to 3D environments. In this regard, we speculate that 3D culture could benefit from scaffolds with higher material stiffness and higher porosity.

The cell line C2C12 is known to be very robust in terms of handling in culture, but recapitulates the steps of myogenesis less accurately (Bach et al., 2004; Maleiner et al., 2018). This should be kept in mind when comparing the behavior of other cell lines with C2C12 cells. Culturing C2C12 cells in the same TE setup (alginate gel scaffolds) as primary murine myoblasts showed that they react similarly to the different culture methods. The effects of changing environments, such as stiffness and degradability of hydrogels, however, were more pronounced in the primary cells, as shown by Boontheekul *et al.* (Boontheekul et al., 2007). Our findings substantiate their observation of limited capability of C2C12 cells to recapitulate the physiological responses in the context of mechanotransduction. Regarding the C25 cell line, it is assumed that their response reflects more closely the situation in non-transformed cells, since transcriptomic analyses have shown that their immortalization did not modifdy any relevant clusters of genes (Thorley et al., 2016).

Overall, we can conclude that the design of an SMTE approach’s material properties is of utmost importance for its ability to promote and foster myogenic differentiation. Particularly evident and relevant from a practical perspective is the observation that cellular behavior observed in 2D setups cannot be translated to more complex 3D structures. The same observation was made concerning the translation of findings from the frequently used murine myoblast line C2C12 to other cell types, specifically the human myoblast line C25. Therefore, our findings contribute to answering the questions of comparability of different approaches in SMTE. Solving this issue, however, will require more attention and studies dedicated to this specific problem in the future.

## Data Availability

The raw data supporting the conclusions of this article will be made available by the authors, without undue reservation.
