# Supplementary material for: Changes in Elastic Moduli of Fibrin Hydrogels Within the Myogenic Range Alter Behavior of Murine C2C12 and Human C25 Myoblasts Differently
Source: Front Bioeng Biotechnol. 2022 May 20;10:836520. doi: 10.3389/fbioe.2022.836520 (PMC9164127; doi:10.3389/fbioe.2022.836520)
Supplement: Supplementary file 1 [file DataSheet1.PDF]

Supplementary Material

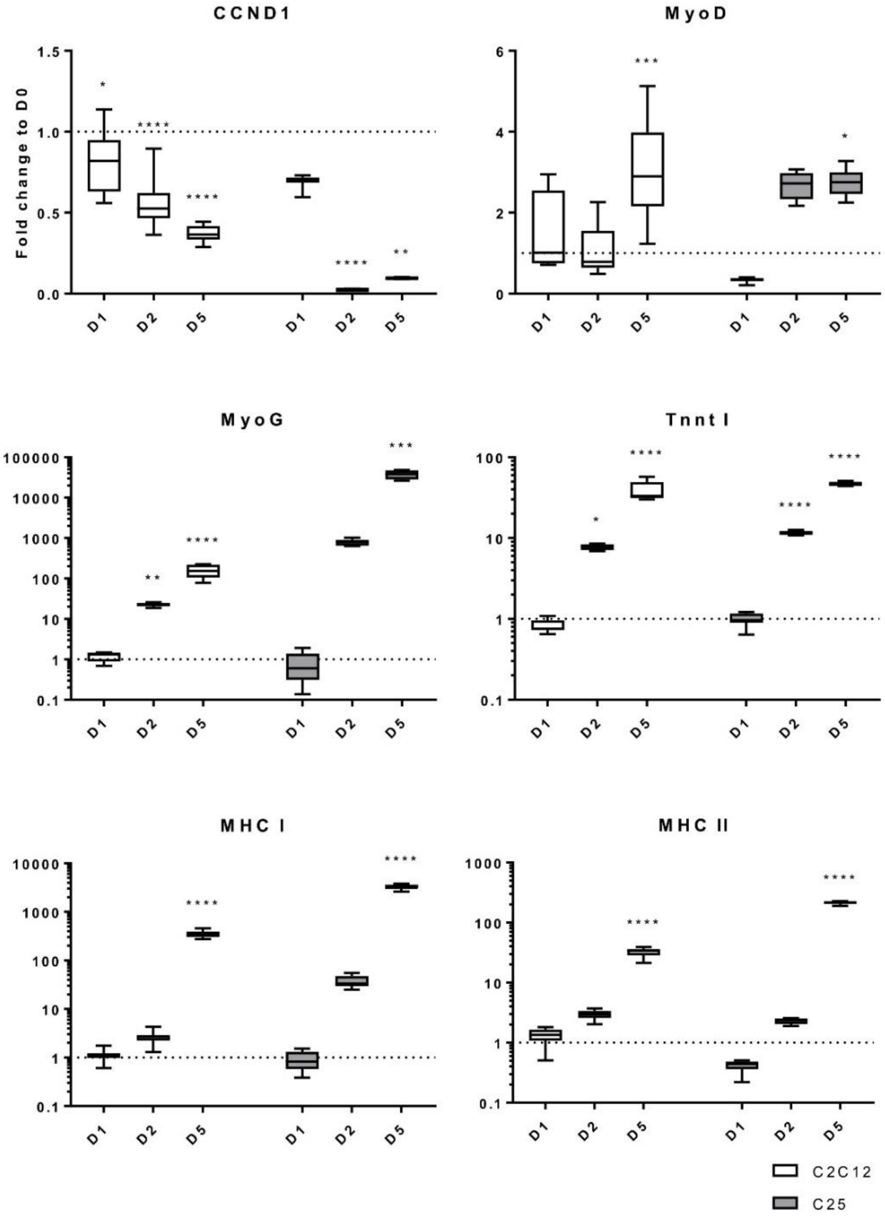

Supplementary figure 1: Myogenic marker gene expression of murine C2C12 and human C25 myoblasts during differentiation. 0.5x10^6 cells were seeded per well of a conventional 6-well plate in growth medium that was replaced by differentiation medium on D1. mRNA expression of marker genes specific for cell cycle progression [*cyclin D 1* (*CCND1*)], early, mid and late stage myogenesis [*MyoD*, *myogenin* (*MyoG*), *troponin T I* (*Tnnt1I*), *myosin heavy chain I* (*MHC I*) and *myosin heavy chain II* (*MHC II*)] was assessed by RT-qPCR. Fold change expression levels were normalized to D0 control samples of each cell line, indicated by the horizontal dotted line; N = 3, n ≥ 7; one-way ANOVA with Sidak's multiple comparison test was performed comparing all time points to D0; *$p < 0.05$, **$p < 0.01$, ***$p < 0.001$, ****$p < 0.0001$.

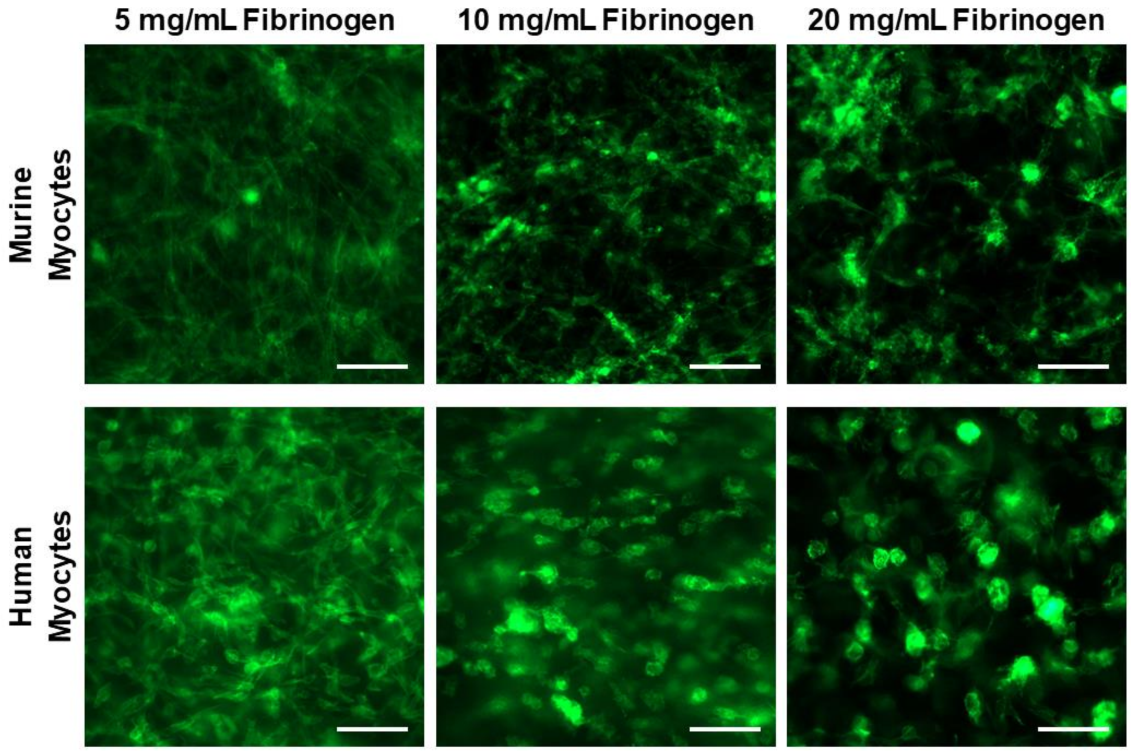

Supplementary figure 2: Myoblasts lose ability to spread when embedded in substrates with higher stiffness. Murine and human myoblasts were cultured embedded in 3D fibrin hydrogels with varying fibrinogen concentrations (5 mg/ml, 10 mg/mL and 20 mg/mL fibrinogen, which correspond to Young´s moduli of 5.1, 10.3 and 20.6 kPa, respectively) under proliferative conditions for 3 days. The cytoskeleton was visualized with phalloidin (green). A representative image is shown for each group (N = 2, n = 4; scale bar represents 100 µm).
